# Role of the Inserted α-Helical Domain in E. coli ATP-Dependent Lon Protease Function

**Published:** 2017

**Authors:** A. M. Kudzhaev, A. G. Andrianova, E. S. Dubovtseva, O. V. Serova, T. V. Rotanova

**Affiliations:** Shemyakin-Ovchinnikov Institute of Bioorganic Chemistry, Russian Academy of Sciences, Miklukho-Maklaya Str., 16/10, Moscow, 117997, Russia

**Keywords:** AAA+ proteins, ATP-dependent proteolysis, DNA binding, inserted α-helical domain, LonA proteases

## Abstract

Multidomain ATP-dependent Lon protease of *E. coli *(Ec-Lon) is one of the key enzymes of the quality control system of the cellular proteome. A recombinant form of Ec-Lon with deletion of the inserted characteristic α-helical HI(CC) domain (Lon-dHI(CC)) has been prepared and investigated to understand the role of this domain. A comparative study of the ATPase, proteolytic, and peptidase activities of the intact Lon protease and Lon-dHI(CC) has been carried out. The ability of the enzymes to undergo autolysis and their ability to bind DNA have been studied as well. It has been shown that the HI(CC) domain of Ec-Lon protease is required for the formation of a functionally active enzyme structure and for the implementation of protein-protein interactions.

## INTRODUCTION

ATP-dependent Lon protease of *Escherichia coli *(Ec- Lon [EC 3.4.21.53], MEROPS: clan SJ, family S16, ID S16.001) is a member of the Lon protease family which plays a key role in the quality control system of the cellular proteome that functions in all domains of life [1- 4]. The Lon family consists of two subfamilies: LonA, which includes bacterial and eukaryotic enzymes, and LonB, which combines the archaea enzymes. Proteases of the subfamilies A and B differ in the domain organization of their subunits, as well as in the environment of the catalytic residues of the proteolytic center [5]. Ec-Lon belongs to subfamily A and degrades abnormal and defective polypeptides, as well as a number of regulatory cellular proteins by a processive mechanism under conditions of a coupling of proteolysis to ATP hydrolysis [4-7]. The distinctive characteristic of Ec- Lon, as well as that of other LonA proteases, is their ability to bind DNA [8-10].

**Fig. 1:**
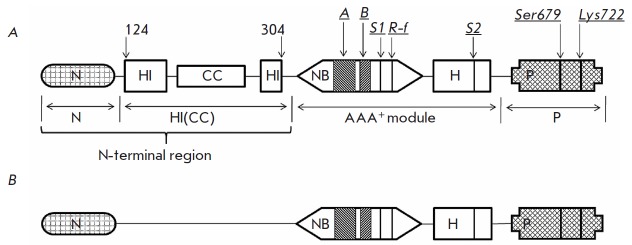
Domain organization of the *E. coli *LonA protease (*A*) and its deletion form Lon-dHI(CC) (*B*). Domain designations: N – N-terminal; HI(CC) – inserted α-helical with a coiled-coil (CC) region; NB – nucleotide-binding; H – α-helical; P – proteolytic. ATPase center components: *A *and *B *– Walker motifs, *S1 *and *S2 *– sensor residues, *R-f *–“arginine finger” residue; proteolytic center components: *Ser679 *and *Lys722 *– catalytic residues.

The Ec-Lon subunit (784 amino acid residues) consists of five domains: N–HI(CC)–NB–H–P (*Fig. 1A*), where the nucleotide-binding (NB) and α-helical (H) domains form a ATPase module that belongs to the superfamily of AAA^+^ proteins (**A**TPases **a**ssociated with various cellular **a**ctivities) [11, 12]; the C-terminal P domain is serine-lysine peptide hydrolase; and the N-terminal and subsequent “inserted” α-helical domains form a non-catalytic region (N-HI(CC)) which includes a sequence fragment with a specific coiled-coil (CC) conformation [13, 14]. The crystal structures of the individual domains (except for the HI(CC) domain) of Ec- Lon and some other LonA proteases have been determined. The spatial structure of the full-length enzymes of the LonA subfamily remains unknown.

The two-domain organization of the N-terminal region is a unique characteristic of Ec-Lon and the entire pool of LonA proteases. LonA proteases differ from other AAA^+^ proteins of the protein quality control system, such as the set of ATP-dependent proteases (ClpAP, ClpXP, FtsH, HslUV) and chaperone-disaggregases (ClpB, Hsp104), by the presence of the inserted HI(CC) domain. We have shown that the HI(CC) domain of Ec-Lon exhibits a marked similarity to both the H domain of its own AAA^+^ module and to the α-helical domain (H1(M)) of the first of the two AAA^+^ modules of ClpB chaperones [13, 14]. At the same time, the role of the HI(CC) domain in the functioning of Ec-Lon protease, its interaction with nucleic acids and/or the structural organization of the enzyme, has not been characterized to date.

In order to study the role of the inserted HI(CC) domain in the manifestation of Ec-Lon functional properties, we performed a comparative study of the enzymatic characteristics and ability to bind DNA of the intact enzyme (*Fig. 1A*) and its deletion form Lon-dHI(CC) without its HI(CC) domain (*Fig. 1B*).

## MATERIALS AND METHODS

**Materials**

Commercial reagents from Sigma, Bio-Rad, Thermo Scientific (USA), Fluka (Switzerland), Boehringer Mannheim (Germany), Pharmacia (Sweden), Difco (England), Panreac (Spain) and Reakhim (Russia) were used in the study.

**Preparation of Ec-Lon (Lon-H_6_) and its deletion form Lon-dHI(CC)**

A recombinant form of Ec-Lon containing a hexahistidine fragment (in LEHHHHHH octapeptide) at the C-terminus of the protein (Lon-H_6_) was prepared according to the previously described procedure [15].

Deletion form Lon-dHI(CC) was obtained on the base of Lon-H_6_ protease. Lon_d_124-304, Lon_HindIII and Lon_BamHI_rev primers (5'-TTTTTTGACCTTGCTGCGCGCATCAATGGTCGGCGACTCCAG-3', 5'-CGCAGAAAGAAGCTTCAACGG-3' and 5'-GTTCTGCTCTGGATCCAGCAC-3', respectively) were constructed using the megaprimer method. Amplification of the gene fragment was carried out in two steps using plasmid DNA pET28-lon-H6 as the template. In the first step, a PCR fragment was obtained using the Lon_d_124-304 and Lon_HindIII primers, and the fragment was subsequently used as the primer in the second step, together with a Lon_BamHI_rev primer. The resulting DNA fragment was about 625 bp in length and was cloned into the pET28_lon vector at the unique HindIII and BamHI restriction sites.

Sequencing of the cloned DNA and synthesis of the primers were carried out by ZAO EVROGEN (www. evrogen.ru). The restriction and ligation procedures were carried out according to the protocols of the manufacturers of the corresponding enzymes.

Isolation and purification of Lon-H_6_ and Lon-dHI(CC) were performed in two steps by Ni^2+^ chelate affinity chromatography using HisTrap FF columns (tandem 2 × 5 mL, GE Healthcare, USA) and anion exchange chromatography on a HiTrap^TM^ Q FF column (5 mL, GE Healthcare) according to the previously described procedure [15].

The protein concentrations were determined by the Bradford method [16].

The homogeneity of the proteins in the preparations was tested electrophoretically [17] using a commercial set of markers (*M*, kDa): β-galactosidase (116.0), bovine serum albumin (66.2), ovalbumin (45.0), lactate dehydrogenase (35.0), Bsp98I restriction enzyme (25.0), β-lactalbumin (18.4), and lysozyme (14.4).

## DNA PURIFICATION

The DNA was purified according to the protocol presented in the manual [18].

**Determination of the enzymatic properties of Lon-H_6_ protease and its deletion form Lon-dHI(CC)**

*ATPase activity *was tested by the accumulation of inorganic phosphate over time in the ATP hydrolysis reaction in 50 mM Tris-HCl buffer, pH 8.1, containing 150 mM NaCl, 5 mM ATP, 20 mM MgCl_2_ and 1 μM enzyme at 37°C [19]. In the control experiment, the enzyme was replaced with a buffer. The initial reaction rates were determined from the optical absorption of a mixture of 200 μL of the reaction medium and 600 μL of the reagent (100 mM Zn(AcO)_2_, 15 mM (NH_4_)_6_Mo_7_O_24_, 1% SDS, pH 4.5–5.0) at a wavelength of 350 nm (ε_350_ = 7,800 M^-1^ cm^-1^).

*The thioesterase activity. *The hydrolysis of thiobenzyl ester of N-substituted tripeptide Suc-Phe-Leu-Phe- SBzl (PepTBE) was monitored spectrophotometrically at a wavelength of 324 nm from the optical absorption of 4-thiopyridone (ε_324_ = 16,500 M^-1^ cm^-1^), which is the product of the reaction between the hydrolysis product (benzylthiolate, BzlS-) and 4,4'-dithiodipyridine (DTDP) [20]. PepTBE hydrolysis was carried out at 37°C in 50 mM Tris-HCl buffer, pH 8.1, containing 150 mM NaCl, 10% DMSO, 0.2 mM DTDP, 0.1 mM PepTBE, and 0.2 μM enzyme. When studying the influence of effectors, a nucleotide up to 2.5 mM and MgCl_2_ up to 20 mM were added to the mixture.

*The proteolytic activity *of the enzymes was tested electrophoretically [17]. The reaction was carried out at a temperature of 37°C in 50 mM Tris-HCl buffer, pH 8.1, containing 150 mM NaCl, 20 μM β-casein and 2–6 μM enzyme, in the absence or presence of 5 mM Nu and 20 mM MgCl_2_. An aliquot of the reaction or control mixture (20 μL) was mixed with 7 μL lysis buffer (0.2 M Tris-HCl, pH 8.9, 4% SDS, 20% glycerol, 0.5 mM EDTA, 0.8% bromophenol blue, 3% mercaptoethanol), refluxed for 10 min, and was applied to a 12% polyacrylamide gel (PAGE) for electrophoresis.

*The autolytic activity *of the enzymes was tested electrophoretically [17] under conditions analogous to the conditions for determining the proteolytic activity, but in the absence of β-casein.

**Testing of the Lon-H_6_ protease and Lon-dHI(CC) protease complexes with plasmid DNA**

The formation of enzyme-DNA complexes was monitored by a deceleration of DNA in an agarose gel (GMSA method) [21]. 20–25 μg of Lon-H_6_ or Lon-dHI(CC) were incubated for 30 min at 25°C with 500 ng of plasmid DNA (pET28a) in 25 μL of 20 mM Tris-HCl buffer, pH 7.5, containing 60 mM NaCl. The protein-DNA complexes were analyzed by gel electrophoresis in a standard 1.0% agarose gel. DNA bands were visualized by staining with ethidium bromide.

## RESULTS AND DISCUSSION

The recombinant Ec-Lon protease used in the study, which contained an additional C-terminal octapeptide bearing a hexahistidine fragment (Lon-H_6_), had been produced and characterized previously [15]. The recombinant deletion form Lon-dHI(CC), without the inserted HI(CC) domain (residues Glu124–Asn304, *Fig. 1B*), was obtained on the base of Lon-H_6_. Preparative amounts of Lon-H_6_ (M 88.5 kDa) and its deletion form Lon-dHI(CC) (M 67.5 kDa) were isolated using affinity chromatography on Ni-Sepharose and anion exchange chromatography on Q Sepharose. A comparative study of the enzymatic activity of intact Lon-H_6_ protease and its deletion form was carried out. Three types of activity were characterized: ATPase, proteolytic (substrate: β-casein), and peptidase (substrate: Suc-Phe-Leu-Phe- SBzl, PepTBE), and the possibility of autolysis of the enzyme preparations was studied. In addition, the presence of nucleic acid in various protein preparations was tested by the phenol extraction method.

**ATPase activity of the deletion form of Ec-Lon protease**

The following standard conditions were selected for testing ATPase activity, as well as other types of activities of Lon-H_6_ protease and its deletion form: 37 °C and 50 mM Tris-HCl buffer, pH 8.1, containing 150 mM NaCl.

It is known that native wt-Ec-Lon exhibits a maximum level of ATPase activity at equal concentrations of ATP and Mg^2+^, and that excess of magnesium ions has an inhibitory effect on the hydrolysis of ATP, which is leveled by binding of the protein substrate [22].

**Fig. 2:**
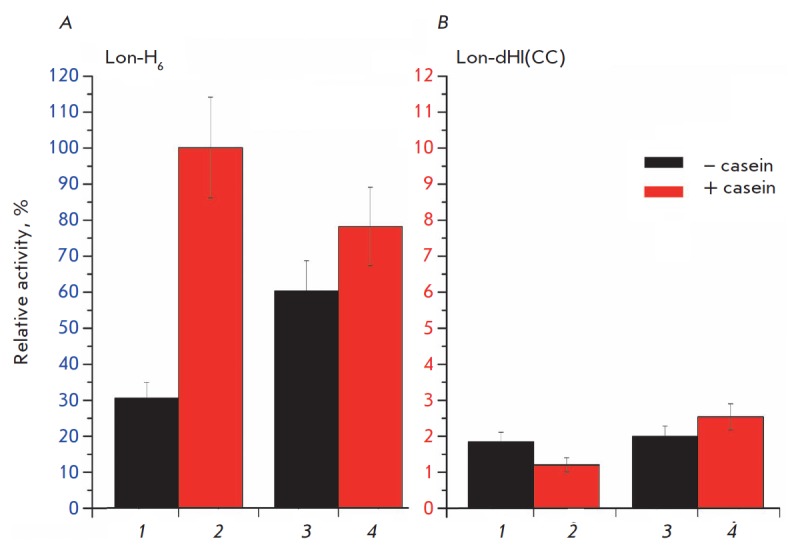
ATPase activity of the intact Lon-H_6_ protease (*A*) and its deletion form Lon-dHI(CC) (*B*) in the absence (black columns) or presence (red columns) of the protein substrate, β-casein. Experimental conditions: 50 mM Tris- HCl buffer, pH 8.1; 0.15 M NaCl; 37°C; concentrations: 5 mM ATP; 20 (*1*, *2*) or 5 mM (*3*, *4*) MgCl_2_; 0 (*1*, *3*) or 0.5 mg/ml (*2*, *4*) β-casein; 0.5-1.0 μM enzyme.

The same trends are typical for intact Lon-H_6_ protease (*Fig. 2A*): the efficiency of hydrolysis of ATP by the enzyme under conditions close to physiological ones (concentration ratio Nu:Mg^2+^ = 1:4) is significantly lower than at equimolar concentrations of Nu and Mg^2+^. Addition of a protein substrate (β-casein) in both cases results in a significant increase in ATPase activity.

The Lon protease almost completely loses its ability to hydrolyze ATP with a loss of the HI(CC) domain: ATPase activity of Lon-dHI(CC) is reduced by more than 10 times compared to the activity of intact Lon-H_6_ protease and by all means does not depend on either the ratio of nucleotide and Mg^2+^ ions concentrations or the addition of a substrate protein (*Fig. 2B*).

The obtained results indicate that the inserted α-helical HI(CC) domain is necessary for the formation of the ATPase center of the Ec-Lon protease and its correct functioning.

**Activity of the peptidase center of the deletion form of Ec-Lon protease**

Similarly to the Lon-H_6_ protease, Lon-dHI(CC) is capable of hydrolyzing a model peptide substrate, PepTBE, but the basic peptidase activity of the deletion form is about 30% that of the activity of the intact enzyme (*Table*). The data in the *Table* demonstrate that only Mg^2+^ ions activate the peptidase centers of both Lon-H_6_ and Lon-dHI(CC). The influence of nucleotide effectors on the intact and modified enzymes is radically different. Free nucleotides (except ADP) and Nu-Mg complexes activate the Lon-H_6_ protease to varying degrees (2–11 times) and ADP inhibits it, but none of the nucleotides has any effect on the hydrolysis of the peptide by Lon-dHI(CC). The Nu-Mg complexes exert a similar but relatively low activating effect on the enzyme peptidase center, comparable to the effect of Mg^2+^ ions. These data show that Lon-dHI(CC) is incapable of binding free nucleotides and weakly interacts with their complexes with magnesium ions. The most powerful effectors affecting the activity of the peptidase center are magnesium ions.

Thus, removal of the HI(CC) domain results in a decrease in the activity of the peptidase center of the Ec- Lon protease and a loss of the regulatory effect of the ATPase center on the peptidase one, which is defined by the nature of the bound nucleotide in the intact enzyme.

**Proteolytic and autolytic activity of the deletion form of Ec-Lon protease**

The proteolytic activity of Lon-H_6_ and its deletion form Lon-dHI(CC) was tested using the hydrolysis of a model protein substrate, β-casein, in the absence and presence of Mg^2+^ ions, free nucleotides, and their complexes. The efficiency of hydrolysis of the target protein and the accumulation of degradation products were detected by gel electrophoresis.

**Table:** Influence of the effectors on the activity of Lon-H_6_ and Lon-dHI(CC) peptidase centers

Effector	Lon-H_6_		Lon-dHI(CC)	
	*v*	*n*	*v*	*n*
No effector	5.88	1	1.64	1
Mg	**33.1**	**5.62**	**5.19**	**3.16**
ATP	**47.1**	**8.01**	1.33	0.81
ADP	0.49	0.08	1.79	1.09
AMPPNP*	**14.2**	**2.41**	1.82	1.11
ATP-Mg	**63.5**	**10.8**	**4.62**	**2.82**
ADP-Mg	**10.1**	**1.73**	**4.89**	**2.98**
AMPPNP-Mg	**58.0**	**9.86**	**5.6**	**3.41**

Note. The specific rates of PepTBE hydrolysis (*v*, ([S], μM)/([E], μM) min) are given; *n *is the ratio of substrate hydrolysis rates in the presence and absence of the effector (*v*_ef_/*v*_0_), where *n * < 1 corresponds to inhibition (*italicized*), and *n *> 1 corresponds to activation of hydrolysis (shown **in bold**). The error did not exceed 10%. Experimental conditions: 50 mM Tris-HCl buffer, pH 8.1; 0.15 M NaCl; 10% DMSO; 0.1 mM PepTBE; 0.2 mM DTDP; 2.5 mM Nu; 20 mM MgCl_2_; 0.2 μM enzyme; 37°C.

^*^ Nonhydrolysable ATP analog, adenosine-5’-(β,γ-imido)triphosphate. ;

**Fig. 3:**
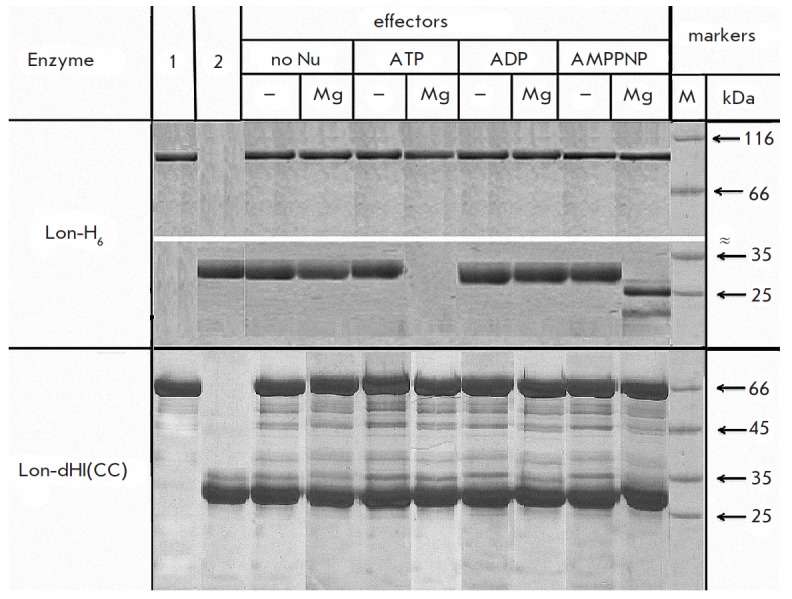
Hydrolysis of β-casein by Lon-H_6_ protease and its deletion form Lon-dHI(CC) with and without effectors (electrophoresis in 12% PAGE). Experimental conditions: 50 mM Tris-HCl buffer, pH 8.1; 0.15 M NaCl; 37°C; reaction time 2 h. Concentrations: Nu – 5 mM; MgCl_2_ – 20 mM; β-casein – 0.5 mg/ml; Lon- H6 – 2.5 μM; Lon-dHI(CC) – 6 μM. 1 – enzyme (control), 2 – β-casein (control), “–“ – in the absence of Mg^2+^, Mg – in the presence of Mg^2+^, M – markers.

The intact Lon-H_6_ protease is capable of hydrolyzing β-casein in two cases: by the processive mechanism (without the formation of large intermediate frag ments) under conditions of a coupling of proteolysis to ATP hydrolysis or by a nonprocessive mechanism in the presence of a complex of a nonhydrolyzable analogue of ATP with magnesium (*Fig. 3*).

Deletion of the HI(CC) domain leads to a complete loss of the proteolytic activity towards β-casein by the deletion form, which indicates the importance of this domain for binding and hydrolyzing the protein substrate (*Fig. 3*). The appearance of bands corresponding to polypeptides with molecular weights ranging from 40 to 60 kDa on the electrophoretic image of the incubated reaction mixture indicates the possibility of self-degradation of Lon-dHI(CC) under the conditions used for the monitoring of the hydrolysis of the target protein.

**Fig. 4:**
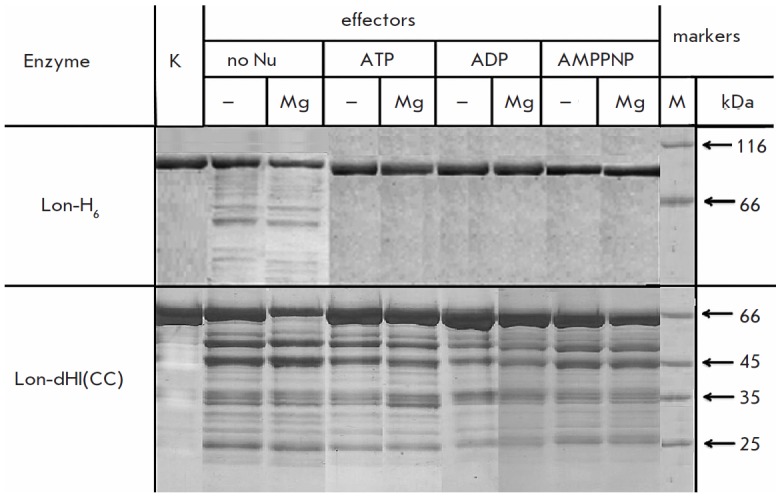
Autolysis of Lon-H_6_ protease and its deletion form Lon-dHI(CC) with and without effectors. The experimental conditions and designations follow *Fig. 3* with the following modifications: Lon-H_6_ – 3.4 μM, reaction time 24 h. K – the original enzyme (control, reaction time 0 h).

Identification of an autolytic activity of Lon-dHI(CC), which accompanies the potential hydrolysis of the protein substrate, required a study of the autolysis process itself. The intact Lon-H_6_ protease was shown to be resistant to self-degradation in the presence of any nucleotide effector (*Fig. 4*). However, during a prolonged incubation (24 hours or more), weak autolysis of Lon-H_6_ is detected in the absence of effectors or in the presence of magnesium ions (*Fig. 4*), which agrees with the previously obtained results [23].

**Fig. 5:**
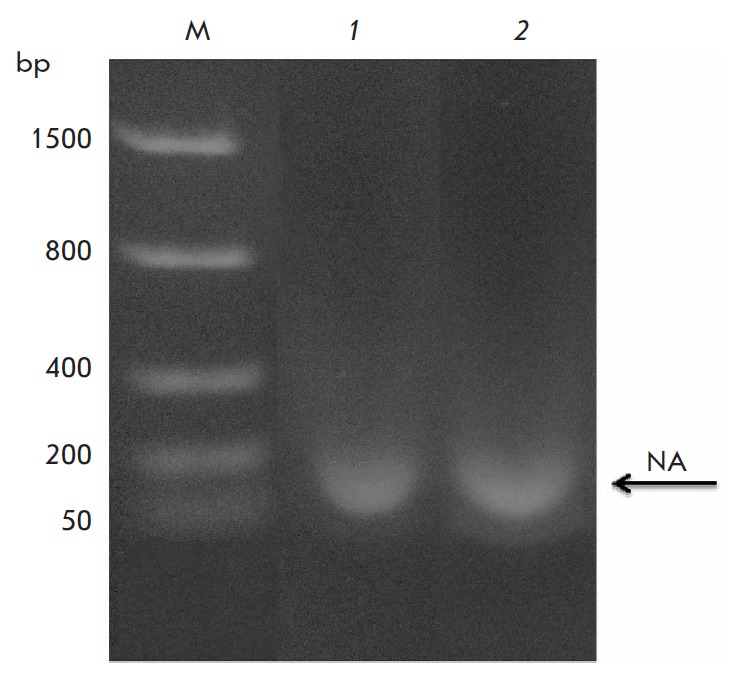
Phenolic extracts of Lon-H_6_ (*1*) and Lon-dHI(CC) (*2*) samples. M *– *markers, NA *– *nucleic acid.

In contrast to Lon-H_6_, the deletion form Lon-dHI(CC) is unstable and it undergoes autolysis both in the absence and presence of nucleotide effectors: moreover, the autolysis of Lon-dHI(CC) is most pronounced in the presence of Mg ions (*Fig. 4*).

**Fig. 6:**
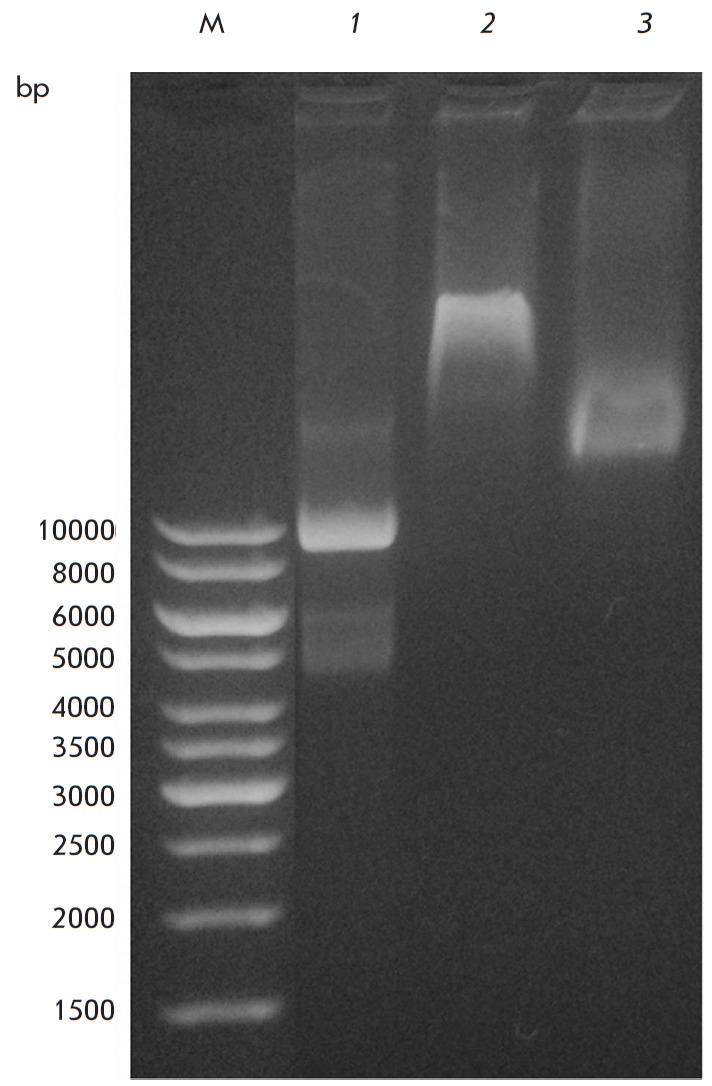
DNA-binding ability of Lon-H_6_ and Lon-dHI(CC). Experimental conditions: 20 mM Tris-HCl buffer, pH 7.5; 60 mM NaCl; 25°C; DNA (pET28a) – 28 nM (*1 – 3*); Lon- H6 – 33.9 μM (*2*), Lon-dHI(CC) – 22.2 μM (*3*); M – markers.

Thus, the loss of the HI(CC) domain leads to a complete loss of the ability of Lon-H_6_ protease to hydrolyze the protein substrate and destabilizes the structure of the enzyme.

**Binding of the nucleic acid by Lon-H_6_ protease and its deletion form Lon-dHI(CC)**

An important characteristic of Ec-Lon is its ability to bind DNA [8-10], but the site of the interaction between the enzyme and nucleic acid has not been localized to date. Since other ATP-dependent proteases of the quality control system of cellular proteins do not have DNA-binding properties and do not contain the characteristic inserted HI(CC) domain typical of LonA proteases, the HI(CC) domain can be expected to be involved in nucleic acid binding. Therefore, we examined the content of nucleic acid in the preparations of Lon-H_6_ protease and its deletion form obtained in the present study.

The DNA content in the preparations of both enzymes, determined from the ratio of optical absorption (A_260_/A_280_) in solutions of Lon-H_6_ and Lon-dHI(CC) (1.09 and 1.06, respectively), did not exceed 5%. The enzyme-bound nucleic acid was isolated from the preparations by the phenol-chloroform extraction method. Treatment of the extracts with benzonase (nonspecific nuclease, Sigma) resulted in exhaustive hydrolysis of the targets, which confirms their classification as nucleic acids. At the same time, both extracts were resistant to treatment with RNase A. These results indicate that both the full-length and deletion forms of Ec-Lon are isolated from *E. coli *cells as complexes with DNA.

Phenol-chloroform extracts were analyzed by electrophoresis in 1% agarose gel, followed by staining with ethidium bromide (*Fig. 5*). It was found that the preparations of both intact Lon-H_6_ protease and Lon-dHI(CC) contain a significant amount of bound DNA in the form of fragments of about 150 bp in size.

In addition, it turned out that both forms of Lon protease are capable of binding additional amounts of nucleic acid. It was shown that the incubation of plasmid DNA with Lon-H_6_ or with Lon-dHI(CC) leads to the formation of DNA enzyme complexes and to a change in the mobility of nucleic acid during electrophoresis in an agarose gel (*Fig. 6*).

The presented data suggest that the HI(CC) domain of the Ec-Lon protease either does not participate in the interaction with nucleic acid or is not determinant in this interaction.

## CONCLUSION

According to the obtained data, the characteristic inserted HI(CC) domain of Ec-Lon protease is necessary for the formation and correct functioning of the enzyme ATPase center. At the same time, the HI(CC) domain does not affect the formation of the peptidase center of Ec-Lon, but it is extremely important for the mutual influence of active sites. It should be emphasized that even though the activity of the peptidase center is retained, deletion of the HI(CC) domain leads to a complete loss of the proteolytic activity of the enzyme, which demonstrates the importance of this domain for the binding and hydrolysis of the protein substrate by Ec-Lon protease.

Interestingly, the deletion forms of the Lon protease from *Brevibacillus thermoruber *(Bt-Lon) [24] without the fragment (246-259) or (248-256) in the coiled-coil (CC) region lose all three types of activity. The discrepancy in the evaluation of the functioning of the peptidase center in the deletion forms of LonA proteases, revealed by comparing the results of this study and the data in [24], may be due to the use of different substrates in the testing of the peptidase center: thiobenzyl ester of the N-protected tripeptide (Suc-Phe-Leu-Phe- SBzl) in our work and 4-methoxy-β-naphthylamide of a less specific tripeptide (Glt-Ala-Ala-Phe-MNA) in [24].

We believe that the identified intensive autolysis of Lon-dHI(CC) is caused by the loss of its ability to efficiently bind nucleotides, a property that is a stabilizing factor for a full-length enzyme. The suggestion that the HI(CC) domain plays the role of a nucleic acid binding site in the Ec-Lon protease has not been experimentally confirmed.

Therefore, it can be concluded that the inserted HI(CC) domain of Ec-Lon-protease is necessary for the formation of a functionally active structure of the enzyme and the implementation of protein-protein interactions.

## Abbreviations

AMPPNP: adenosine 5′-(β,γ-imido)triphosphate
DTDP: 4,4′-dithiodipyridine
Glt: glutaryl
Nu: nucleotide
PepTBE: Suc-Phe-Leu-Phe-SBzl
Suc: succinyl

## References

[1] Gottesman S., Wickner S., Maurizi M.R. (1997). Genes Dev..

[2] Tyedmers J., Mogk A., Bukau B. (2010). Nat. Rev. Mol. Cell. Biol..

[3] Mogk A., Haslberger T., Tessarz P., Bukau B. (2008). Biochem. Soc. Trans..

[4] Lee I., Suzuki C.K. (2008). Biochim. Biophys. Acta..

[5] Rotanova T.V., Melnikov E.E., Khalatova A.G., Makhovskaya O.V., Botos I., Wlodawer A., Gustchina A. (2004). Eur. J. Biochem..

[6] Goldberg A.L., Moerschell R.P., Chung C.H., Maurizi M.R. (1994). Meth. Enzymol..

[7] Charette M.F., Henderson G.W., Markovitz A. (1981). Proc. Natl. Acad. Sci. USA..

[8] Fu G.K., Smith M.J., Markovitz D.M. (1997). J. Biol. Chem..

[9] Lee A.Y.L., Hsu C.H., Wu S.H. (2004). J. Biol. Chem..

[10] Liu T., Lu B., Lee I., Ondrovicova G., Kutejova E., Suzuki C.K. (2004). J. Biol. Chem..

[11] Lupas A.N., Martin J. (2002). Curr. Opin. Struct. Biol..

[12] Iyer L.M., Leipe D.D., Koonin E.V., Aravind L. (2004). J. Struct. Biol..

[13] Rotanova T.V., Melnikov E.E. (2010). Biochemistry (Moscow) Suppl. Series B: Biomed. Chem..

[14] Rotanova T.V., Dergousova N.I., Morozkin A.D. (2013). Russ. J. Bioorgan. Chem..

[15] Andrianova A.G., Kudzhaev A.M., Serova O.V., Dergousova N.I., Rotanova T.V. (2014). Russ. J. Bioorgan. Chem..

[16] Bradford M.M. (1976). Anal. Biochem..

[17] Laemmli U.K. (1970). Nature.

[18] Maniatis T., Fritsch E.F., Sambrook J. (1982). Molecular Cloning: A Laboratory Manual. Cold Spring Harbor Laboratory Press, Cold Spring Harbor, NY, 1982..

[19] Bencini D.A., Wild J.R., O’Donovan G.A. (1983). Anal. Biochem..

[20] Castillo M.J., Nakajima K., Zimmerman M., Powers J.C. (1979). Anal. Biochem..

[21] Lee A.Y.L., Tsay S.S., Chen M.Y., Wu S.H. (2004). Eur. J. Biochem..

[22] Melnikov E.E., Tsirulnikov K.B., Rotanova T.V. (2000). Russ. J. Bioorgan. Chem..

[23] Kudzhaev A.M., Andrianova A.G., Serova O.V., Arkhipova V.A., Dubovtseva E.S., Rotanova T.V. (2015). Russ. J. Bioorgan. Chem..

[24] Chir J.L., Liao J.H., Lin Y.C., Wu S.H. (2009). Biochem. Biophys. Res. Commun..

